# Hemostasis in uncontrolled esophageal variceal bleeding by self−expanding metal stents: a systematic review 

**Published:** 2016

**Authors:** Stefano Pontone, Michela Giusto, Angelo Filippini, Clelia Cicerone, Daniele Pironi, Manuela Merli

**Affiliations:** 1 *Department of Surgical Sciences, “Sapienza” University of Rome, Italy*; 2 *Department of Clinical Medicine, “Sapienza” University of Rome, Italy*; 3 *Department of Internal Medicine and Medical Specialties, “Sapienza” University of Rome, Italy*

**Keywords:** Nonselective β-blockers, TIPSS, Endoscopic band ligation, Uncontrolled bleeding, Self-expanding metal stent

## Abstract

**Aim:**

The aim of this systematic review was to evaluate the current reported efficacy and the mortality rate of SEMS treatment in uncontrolled bleeding patients.

**Background:**

Esophageal variceal bleeding (EVB) represents a life threatening pathology. Despite the adequate pharmacologic and endoscopic treatment, continuous or recurrent bleeding, named as uncontrolled bleeding, occurs in 10-20% of cases. A new removable, covered, and self-expanding metal stent (SEMS) was proposed to control the variceal bleeding.

**Materials and methods:**

The study was conducted according to the PRISMA statement. Studies were identified by searching MEDLINE (1989-present) and SCOPUS (1989-present) databases. The last search was run on 01 July 2015.

**Results:**

Nine studies (period range=2002-2015) met the inclusion criteria and were included in quantitative analysis. High rate of SEMS efficacy in controling acute bleeding was observed, with a reported percentage ranging from 77.7 to 100%. In 10% to 20% of patients, re-bleeding occurred with SEMS in situ. Stent deployment was successful in 77.8% to 100% of patients while 11 to 36.5% of patients experienced stent migration.

**Conclusion:**

SEMS could be effective and safe in control EVB and can be proposed as a reliable option to ballon tamponed for patient stabilization and as a bridging to other therapeutic approach.

## Introduction

 Esophageal variceal bleeding (EVB) represents a life threatening pathology associated with a six-week mortality rate of 20% following the initial bleeding episode (1, 2). Prevention of re-bleeding can be achieved using non-selective β-blockers (NSBBs), endoscopic band ligation (EBL), a combination of NSBBs and EBL, as well as transjugular intrahepatic portosystemic stent shunt (TIPSS). Despite the adequate pharmacologic and endoscopic treatment, continuous or recurrent bleeding, named as uncontrolled bleeding, occurs in 10-20% of cases (3). Sengstaken-blakemore balloon tamponade (BT), may control the initial variceal hemorrhage in > 80% of patients, leading to a high complication rate (4, 5). Early TIPSS placement and related technical improvements (6, 7) reduced treatment failure in these patients. The current available techniques in the event of uncontrolled bleeding are not getting enough. Recently, a new treatment option, represented by removable, covered, self-expanding metal stent (SEMS) was suggested to control acute refractory variceal bleeding (8). Recently, SEMS have been proposed as a safer option than BT in uncontrolled esophageal variceal bleeding (3) even if the level of evidence is still low since data derive mainly from case-series. 

The aim of this systematic review was to collect all the currently available studies utilizing SEMS for the treatment of acute esophageal variceal bleeding as the first line therapy or as a rescue therapy in order to asses efficacy and feasibility of SEMS in control bleeding and their safety. 

## Material and Methods

**Figure 1:**
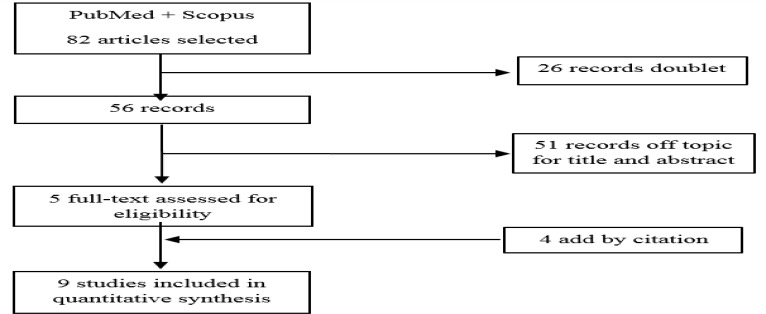
Flow chart of systematic review.

The study was conducted according to the PRISMA statement (9). We included only English uncontrolled esophageal variceal bleeding. No publication date or publication study restrictions were imposed. Participants of any age and sex were considered. The primary outcome measure was the correct positioning rate, and the secondary outcome was the failure to control bleeding rate as defined in the Baveno IV Consensus Workshop (10). We excluded abstracts, review articles, meta-analyses, and editorials. Studies were identified by searching Pubmed (1989-present) and SCOPUS (1989-present) databases. The last search was run on 01 July 2015. Search criteria are summarized in Figure 1. We used the following search terms for all databases: “variceal bleeding SEMS; AND/OR? oesophageal stent variceal bleeding”. Eligibility assessment was performed independently in a blinded standardized manner by four reviewers. One review author extracted the following data from included studies: type of study, the number of participants, primary and secondary outcomes, as well as a diagnostic tool used. A senior investigator resolved disagreements between reviewers. The information extracted were: reference with acquisition data range year, study classification, number of patients enrolled, SEMS type, the presence of active bleeding, effectiveness of immediate hemostasis and rebleeding/migration rate (Table 1). 

## Results

**Study selection**

The search in PubMed and Scopus provided a total of 82 citations. After adjusting for duplicates, 56 articles remained. Of these, 51 were discarded because the title and/or the abstract were off-topic. With respect to the remaining five studies, it appears necessary to add four articles by citations. Finally, nine studies met the inclusion criteria and were included in the analysis (Figure 1) (11-19).

**Study characteristics**

All selected papers were single center case series and most of them belonged to the European area. Almost all studies employed SEMS as a rescue therapy after failure of combined endoscopy and pharmacology treatments or even after BT. The data collection period range was 2002-2014. Although many papers on uncontrolled bleeding have been published in the US, the studies concerning SEMS were performed in Austria (16, 17), Egypt (11), Netherlands (13), Moldavia (18) and Germany (19). 

**Patients’ characteristics**

One hundred twenty four patients were enrolled into the nine studies selected for review (92 male; mean age 56 years; age range 18-87). All the studies included cirrhotic patients with at least Child B cirrhosis, except for two patients with Child-Pugh score A. Median Meld was 27 (range 8-40). The main inclusion criterion was the presence of uncontrolled esophageal varices bleeding with or without BT before stenting insertion. 

**SEMS characteristics and related data**

Excluding the Hubmann study (16), in which Choo Stent NES−18−080−070 (M.I. Tech Co., Ltd) and Boubela-Danis esophageal stents were used, most studies used the SX-Ella Danis stent (Ella-CS, Hradec Kralove, Czech Republic) as a removable, covered, and self-expanding metal stent to treat uncontrolled bleeding. This SEMS can be deployed in the lower esophagus without traditional radiological or endoscopic assistance. Two studies (11, 18) reported the amount of time spent for stent application, which was about 4-10 minutes. A high rate of SEMS efficacy to control acute bleeding was observed, with a reported percentage ranging from 77.7 to 100%. In 10% to 20% of patients re-bleeding occurred, even though SEMS was correctly in place. Stent deployment was successful in 77.8% to 100% of patients while 11 to 80% of patients experienced stent migration. Stent remained “in situ” for a few hours to 214 days. The migration of the stent was not related to the re-bleeding. Intra-procedural mortality was not reported. Severe adverse complications were extremely rare, while the main complication reported at removal was the development of esophagus distal ulcerations. 

## Discussion

Combined treatment with vasoactive drugs, prophylactic antibiotics, endoscopic techniques, and together with hemodynamic resuscitation, is the recommended standard care for patients with acute variceal bleeding (8). However, treatment failure occurs in about 10 to 15% of patients. Management of failures includes repeated endoscopic treatment. Moreover, in most severe patients, TIPSS placement has to be considered as a rescue therapy (8). Although TIPSS insertion is highly effective with control of bleeding, it is still complicated by high mortality mainly due to a worsening in liver function (24). For that reason, an early TIPSS placement, within 72 hours (ideally <24 hours) is suggested in patients with variceal bleeding and at high-risk of treatment failure (8, 25-27) since it has been shown to not only prevented recurrent bleeding but also improved survival (25, 26). However, until definitive treatment can be instituted, temporary “bridge treatments” need to be applied in patients who present an uncontrolled bleeding. 

**Table 1:** Characteristics of the included studies using Self Expandable metal stents (SEMS)=SX-ELLA Stent Danis

**Author** **Study period** **Geographic Area**	**Classification**	**Patients Characteristics**	**Number of Active Bleedings**	**Treatment approach** ** prior to stenting**	**Number of Immediate ** **bleeding controls**	**Number of Rebleeding ** **events with** **stent “in situ”**	**Stent ** **Duration** **(days,range)**	**Number of** **Successful** **Stent** **Deployment**	**Number of** **Migration/** **Complications at removal**
Hubmann R 2002-2005 Austria	Case Series* Single center	20 pts (18M) Median Age 52 yrs (27-87) Child B: 8; Child C:12	20	Numbers of BL=11 Numbers of BL+ST=5 Numbers of BL+BT=1	20 (100%)	0	1-14	20 (100%)	5 (25%)**/1 small ulceration in the distal esophagus
Zehetner J 2003-2006 Austria	Case Series Single center	34 pts Median Age 56 yrs (32-91) Child B 13; Child C: 21	34	Numbers of BL=21 Numbers of ST=8 Numbers of BT: 6	34 (100%)	0	1-14	Not reported	7 (20.5%)/1 slight ulceration in the distal esophagus
Wright G 2007-2008 UK	Case Series Single center	10 pts (9M) Median Age 49,6 yrs (14-39) Median MELD score25 (14-39)	9	Numbers of endoscopic hemostasis treatments =5 Numbers of BT=3	7 (77.8%)	1 (10%)	6-14	9 (100%)	2 (22.2%) Complicated with esophageal Perforation/1 ulceration in the distal esophagus
Dechêne A 2007-2011 Germany	Case Series Single center	8 pts (6M) Mean Age 63±11 Median MELD score31 (16-41) Child C=8	9	Numbers of BL=6 Numbers of ST+BL+BT=2	9 (100%)	0	7-14	9 (100%)	1 (11%)/none
Zahkaria MS 2008-2009 Egypt	Case Series Single center	16pts (14M) Mean Age 56±6 Child A=2, Child B=8; Child C=6	16	None	14 (87.5%)	0	2-4	15 (93.7%)	6 (36.5%)/1 deep ulceration in the distal esophagus
Fierz FC 2010-2011 Switzerland	Case Series Multicentric	7pts (5M) Median Age56 yrs (41-68) Child B=2; Child C=5 Median MELD score29 (11-37)	9	Numbers of BL=4 Numbers of BL+ST=2	8 (88.9%)	0	12 h-5 days	7 (77.8%)	2 (22.2%)/none
Ghidirim Gh.P 2010-2012 Moldavia	Case series Single center	14 pts (8M) Median Age 51 (range 32-69) Mean Child: 9.5±0.4 Mean MELD score: 17.7±1.7	9	Number of BL=14	14 (100%)	0	18h-7 days	14 (100%)	5 41.6%/not reported
Holster IL 2012 Netherlands	Case Series Single center	5 pts (3M) Median Age 58 yrs (48-78) Median MELD score 21 (11-28)	5	Numbers of BL=5	5 (100%)	1 (20%)	6-214	Not reported	1 (20%)/None
Mὒller M 2011-2014 Germany	Case Series Single center	10 pts (8M); Median Age: 6 (range 43-79) Child B=6; Child C=4 Median MELD Score 15.5 (8-27)	1	Not reported	10 (100%)	0	5-24 days	10 (100%)	8 (80%)/ 2 ulcerations

Band Ligation=BT; Sclerotherapy: ST; Ballon Tamponade:BT;

* Two patients with Choo stents, three patients Ella±Boubela stents, and 15 patients with Ella±Danis stents;

** One patients with Choo stent, two patients with Ella±Boubela stents, and two patients with Ella±Danis stents

The BT, performed by skilled specialists, is an effective method to control the acute variceal bleeding in achieving hemostasis in 61% of patients (20). On the other hand, the BT can be adopted as a life-saving procedure by non-specialists in unprotected areas. This may explain the high rate of complications and the relatively low rate of effectiveness. 

However, considering the high rate of possible serious complications caused by the blind technique, the use of BT has recently been discouraged in the last Baveno consensus (8). For this reason, other inserting techniques (21) and devices were proposed.

Currently, a promising alternative is represented by covered SEMS. Since 2002 until today, nine studies examined the efficacy and safety of stents as treatment for uncontrolled bleeding. This method follows the rationale of BT exploiting the potential of the stent to compress varices with the radial force as initially used for palliation of malignant stenosis. Although the first prototype needed the radiological guide during positioning, a subsequent development bypassed this disadvantage using a specific insertion device (16).

Waiting for the next publication of randomized multicenter studies (22), available data suggest that SEMS placement allows a safe bridging from the acute bleeding episode to the therapeutic procedures, being able to induce an effective control of the esophageal variceal bleeding (23). However, stent deployment may be problematic and stent migrations may occur. Furthermore, removal of the stent may lead to the development of ulceration of the distal esophagus. Learning curve for the endoscopy placement and removal of the stents has not been reported, so if an increasing specialist skill would lead to an increase in the rate of correct stents’ placement and a reduction of stents’ migration remains an open question. 

In conclusions, SEMS is effective and safe in control EVB and can be proposed as a reliable option to BT for patient’s stabilization and as a bridging to other therapeutic approach. Further studies, including a large number of consecutive patients and non-specialists performed procedures are needed to increase the reliability of the reported data.
